# Fast and Easy Nanopore Sequencing Workflow for Rapid Genetic Testing of Familial Hypercholesterolemia

**DOI:** 10.3389/fgene.2022.836231

**Published:** 2022-02-09

**Authors:** Muhidien Soufi, Simon Bedenbender, Volker Ruppert, Bilgen Kurt, Bernhard Schieffer, Juergen R. Schaefer

**Affiliations:** ^1^ Center for Undiagnosed and Rare Diseases, University Hospital Giessen and Marburg and Philipps University Marburg, Marburg, Germany; ^2^ Department of Cardiology, Angiology and Critical Care Medicine, University Hospital Giessen and Marburg and Philipps University Marburg, Marburg, Germany

**Keywords:** familial hypercholesterolemia, LDL receptor, rapid genetic testing, oxford nanopore sequencing, long amplicon sequencing, genetic diagnosis, hereditary diseases

## Abstract

Familial hypercholesterolemia (FH) is an autosomal dominant lipid metabolism disorder characterized by severely elevated plasma low-density lipoprotein cholesterol levels. The disease is caused by mutations in 3 genes (*LDLR*, *APOB* and *PCSK9*) while over 90% of the mutations are located within the *LDLR* gene. Thus, genetic analysis of the *LDLR* gene is the first step in the genetic diagnosis of FH. However, conventional methods like Sanger and NextGen sequencing are still costly and time-consuming. In contrast, Oxford Nanopore technology sequencing is an emerging third-generation sequencing technology featured by easy operability, low cost, small size and the capability of parallel sample sequencing. Here, we present an easy Nanopore-sequencing-based workflow for the rapid genetic testing of FH taking only 3 days and costing less than $50 per sample without the requirement for deep bioinformatic knowledge. Using our workflow, we were able to identify the underlying pathogenic variants of 10 FH patients including one novel, not yet recorded pathogenic variants. Our workflow allows the rapid evaluation of the pathogenic variants by utilizing detailed variant information from Ensembl. Additionally, our workflow is not restricted to sequencing the *LDLR* gene alone but can be easily adapted to the other FH-causing genes and more importantly, to any desired gene contributing to any hereditary disease. Therefore, our workflow is an attractive opportunity for every diagnostic laboratory to offer fast and easy in-house genetic diagnostics.

## Introduction

Familial hypercholesterolemia (FH) is an autosomal dominant disorder of lipid metabolism characterized by highly elevated plasma levels of low-density lipoprotein cholesterol (LDL-C). Affected individuals have a high risk for the development of premature atherosclerosis and early onset coronary artery disease due to impaired clearance and accumulation of low-density lipoprotein (LDL) particles in the cardiovascular system (Youngblom et al., 1993; Defesche et al., 2017; Alonso et al., 2018). The estimated prevalence of heterozygous FH in European populations is 1/500 but recent studies suggest a higher prevalence up to 1/200 (Youngblom et al., 1993; Nordestgaard et al., 2013; Defesche et al., 2017). Individuals with heterozygous FH have markedly elevated plasma LDL-C levels and if not adequately treated develop coronary artery disease within the third decade of their live. Homozygous FH is a very rare condition with a prevalence of 1/1,000,000 in the general population. Similarly, recent studies estimate a much higher prevalence of 1/300,000 (Defesche et al., 2017; Benito-Vicente et al., 2018). Homozygous individuals have extremely elevated plasma LDL-C levels and develop xanthomas, carotid artery stenosis and aortic valve stenosis in the first decade of life. Without intervention in early childhood, these individuals usually die within the first 2 decades of their lives (Youngblom et al., 1993). The molecular basis for FH are mutations in one of three genes: low-density lipoprotein receptor (*LDLR*), apolipoprotein B (*APOB*) and protein convertase subtilisin/kexin type 9 (*PCSK9*). The LDLR is responsible for the receptor-mediated endocytosis of LDL particles which consist of APOB, lipids and cholesterol whereas PCSK9 controls the amount of LDLR on the cell surface by increasing LDLR degradation (Benito-Vicente et al., 2018; Roth and Davidson, 2018). The LDLR is expressed mainly in hepatocytes and accounts for the clearance of 70% of all plasma circulating LDL (Brown and Goldstein, 1986). Studies on the prevalence of mutations within these 3 genes showed that the vast majority (93% in UK population (Humphries et al., 2006)) of mutations is located in the *LDLR* gene. To date, more than 3,000 variants within the *LDLR* gene have been identified and reported in ClinVar (Landrum et al., 2018). According to their position within the coding sequence, FH-causing LDLR variants can be categorized into five classes: class 1 (no receptor synthesis), class 2 (impaired intracellular receptor transport), class 3 (defective LDL binding by the receptor), class 4 (defective receptor internalization) and class 5 (impaired receptor recycling) (Hobbs et al., 1990).

Given that the majority of FH cases are associated with pathogenic variants in the *LDLR* gene, genetic testing by sequencing of the entire *LDLR* gene is the first step in genetic diagnosis of FH. However, conventional Sanger sequencing of PCR amplicons still is challenging, costly and time consuming for many routine diagnostic laboratories, especially in cases when numerous FH samples need to be sequenced. Oxford Nanopore technology (ONT) sequencing is a third-generation sequencing technology that enables long-read sequencing in real time. Nanopore sequencing measures the change of an ion current when the DNA is passed through a nanopore by a motor enzyme. This sequence-dependent change in ion current is then used to determine the bases of the analyzed sequence which is called basecalling (Jain et al., 2016; Rang et al., 2018). The ONT MiniON Mk1C device is an easy to operate, low cost and small size sequencing device that allows sequencing of multiple samples in parallel. Due to its low computational system requirements, the ONT MinION sequencing platform is a cost-effective alternative for every diagnostic laboratory (Jain et al., 2016; Petersen et al., 2019).

In this report, we describe a Nanopore sequencing for the genetic analysis of the *LDLR* gene with the ONT MinION Mk1C sequencing device protocol using long-amplicon sequencing with rapid barcoding. Our protocol is fast, easy and allows sequencing of multiple FH samples in parallel. Our workflow includes a simple bioinformatic analysis workflow, which does not require any programming skills to rapidly identify the *LDLR* variants. The results allow fast evaluation of the variants by using information about clinical significance, variant consequence as well as SIFT and PolyPhen2 predictions.

## Materials and Methods

### Patients

For validation experiments of this study, 4 patients (3 male and 1 female) aged 2–36 years were included who had a genetically proven diagnosis of homozygous or heterozygous FH and known disease-causing *LDLR* variants verified by Sanger sequencing. The patient characteristics and ethnicities are stated in Table 1.

**TABLE 1:** Characteristic of patients selected for the validation experiments and workflow results.

patient ID	1	2	3	4
patient characteristics				
age (years)	36	2	32	23
sex	M	M	M	F
ethnicity	Turkish	Turkish	Turkish	Turkish
TC (mmol/L)	9.18	10.83	8.90	17.93
LDL-C (mmol/L)	7.94	9.18	5.57	16.26
genetic change	c.1729T>C	c.761A>C	c.761A>C	c.1567G>A
exon	12	5	5	10
amino acid change	p.(Trp577Arg)	p.(Gln254Pro)	p.(Gln254Pro)	p.(Val523Met)
protein consequence	missense variant	missense variant	missense variant	missense variant
zygosity (%)	heterozygous (39.3)	homozygous (88.8)	heterozygous (48.4)	homozygous (91.5)
dbSNP reference	rs8792550000	rs879254667	rs879254667	rs28942080
variant calling summary				
total variants	36	22	26	33
recorded in dbSNP	31	16	20	25
not recorded in dbSNP	5	6	6	8
variants in CDS	5	2	3	5
variants in non-CDS	31	20	23	28

TC, total cholesterol; LDL-C, low-density lipoprotein cholesterol; CDS, coding sequence.

For the identification of unknown pathogenic variants, 3 patients from a single family (2 male and 1 female) aged 6–40 years and 3 additional unrelated female patients aged 5–43 years with plasma LDL concentration ranging from 5.8 to 18.7 mmol/L and suspected FH based on characteristic symptoms including xanthomas and a familial history of premature coronary artery disease were included. The patient characteristics and ethnicities are stated in Table 2.

**TABLE 2:** Identification of unknown variants in patients with suspected FH.

patient ID	5	6	7	8	9	10
age	38	6	40	44	13	6
sex	F	M	M	F	F	F
ethnicity	German	German	German	German	Turkish	Lebanese
TC (mmol/L)	7.62	14.86	7.88	12.67	14.86	20.61
LDL-C (mmol/L)	5.81	13.52	6.46	9.59	13.26	18.69
genetic change	c.1916T>A	c.1916T>A c.1844A>G	c.1844A>G	c.653del	c.1474G>A	c.1729T>C
exon	13	13/12	12	4	10	12
amino acid change	p.(Val639Asp)	p.(Val639Asp) p.(Glu615Gly)	p.(Glu615Gly)	p.(Gly218ValfsTer47)	p.(Asp492Asn)	p.(Trp577Arg)
protein consequence	missense variant	missense variant	missense variant	frame shift	missense variant	missense variant
zygosity (%)	heterozygous (46.2)	compound heterozygous (49.7/46.6)	heterozygous (46.0)	heterozygous (48.5)	homozygous (95.4)	homozygous (86.6)
dbSNP reference	rs794728584	rs794728584/–	—	rs137853966	rs373646964	rs879255000
pathogenicity	likely pathogenic	likely pathogenic/–	—	pathogenic	pathogenic	pathogenic
SIFT Sim et al. (2012)	deleterious	deleterious/deleterious	deleterious	—	deleterious	deleterious
polyphen2 Adzhubei et al. (2010)	benign	benign/probably damaging	probably damaging	—	probably damaging	probably damaging
PROVEAN protein Choi and Chan (2015)	deleterious	deleterious/deleterious	deleterious	—	deleterious	deleterious
mutation-taster Steinhaus et al. (2021)	disease causing	disease causing/disease causing	disease causing	disease causing	disease causing	disease causing

TC, total cholesterol; LDL-C, low-density lipoprotein cholesterol.

All patients attended the Lipids Competence Center of the Department of Cardiology at the University Hospital Marburg and gave written informed consent for genetic analysis. All procedures were in accordance with the Helsinki Declaration of 1975, as revised in 1996 and the local ethics committee at the Philipps-University, Marburg.

### Genomic DNA Isolation and PCR Amplification

Genomic DNA was isolated from whole EDTA blood using the Quick-DNA HMW MagBead Kit (Zymo Research, Freiburg, Germany). The *LDLR* gene was amplified in 5 fragments spanning the promoter region and all 18 exons of the *LDLR* gene (Supplementary Table S1). Touchdown PCR reactions were performed in 25 µl reactions containing 12.5 µL LongAmp Taq 2X Master Mix (New England BioLabs, Frankfurt, Germany), 1 µM of each forward primer and reverse primer and 160 ng genomic DNA. PCR cycling conditions were: Initial denaturation for 30 s at 94°C; 20 cycles of 30 s denaturation at 94°C, 30 s annealing at 63–58°C (-0.5°C/2 cycles), 7 min 30 s extension at 65°C; followed by 30 cycles of denaturation at 94°C, 30 s annealing at 58°, 7 min 30 s extension at 65°C and 10 min final extension at 65°C. Purity and correct sizes of the amplicons were verified by DNA agarose gel electrophoresis.

### Oxford Nanopore Sequencing

5 µl of each PCR product were treated with the Exo-CIP Rapid PCR Cleanup Kit (New England BioLabs, Frankfurt, Germany). 100 fmol of each fragment were pooled (final volume 25 µl) and used subsequently for library preparation with the SQK-RBK110.96 rapid barcoding kit (Oxford Nanopore Technologies, Oxford, United Kingdom). For addition of rapid barcodes, 2.5 µL of rapid barcode mix and 2.5 µL of nuclease free water were added to 5 µL of the pooled fragment mix. The rapid barcoding reaction mixes were incubated at 30°C for 2 min followed by 2 min at 80°C. Subsequent cleanup, priming and loading of the MinION flow cells (FLO-MIN106D) were performed according to the manufactures protocol. For sequencing with a Flongle flow cell (FLO-FLG001), half of the volumes for addition of rapid barcodes were used. Sequencing runs were performed on a MinION Mk1C instrument (Oxford Nanopore Technologies, Oxford, United Kingdom).

### Bioinformatic Analysis

Bioinformatic analysis was performed with Geneious Prime 2022.0.1 (https://www.geneious.com). Barcoded raw reads were mapped against the *LDLR* reference sequence (NG_009060.1) using the MiniMap2 plugin (Li, 2018). Variant calling was performed using the implemented Variations/SNP caller. Found variants were assigned to recorded variants in Ensembl by the annotation comparison tool (for the detailed procedure please refer to the guideline in the Supplementary Material).

## Results

### Validation of the Workflow

**FIGURE 1:**
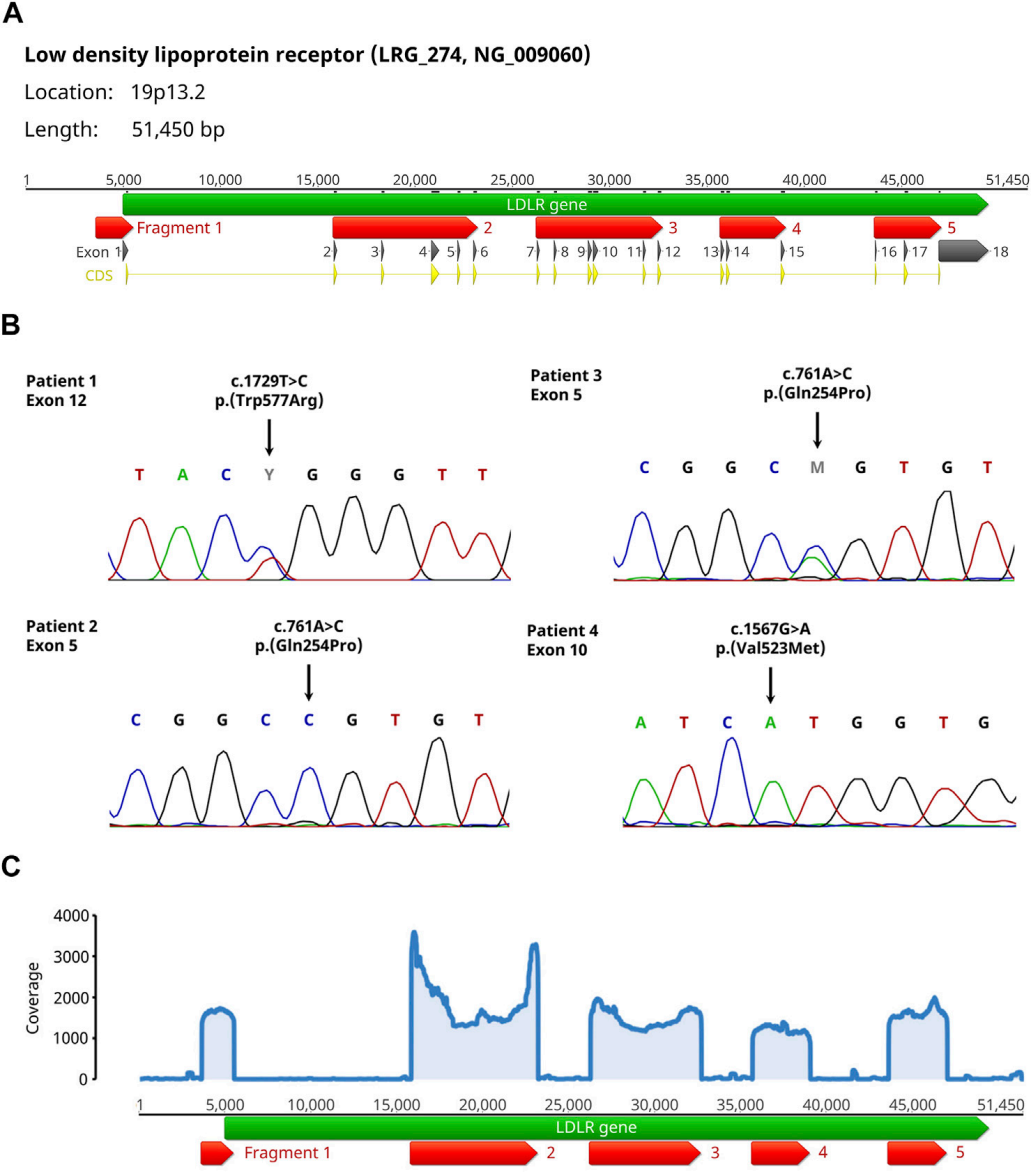
Setup and results of the validation experiments for the Nanopore sequencing workflow of the *LDLR* gene. **(A)** The *LDLR* gene (green arrow) is amplified in 5 fragments (red arrows) covering the promoter region and the coding sequences (yellow arrows) of all 18 exons (dark grey arrows). Please note, that fragment 5 only covers the starting sequence of exon 18 because the remaining part contains only untranslated sequences. **(B)** Sanger sequencing results from the 4 patients with homo- or heterozygous FH showing the pathogenic variants within Exons 5, 10 and 12 including the change on DNA and protein level. **(C)** Coverage plot of the individual fragments showing equal distribution of coverage among all fragments exemplary for patient 4. For all 4 patients, the mean coverage was above 900 with a maximum mean coverage of 2,347.

We amplified the *LDLR* gene in 5 fragments covering the promoter region and the coding sequences of all 18 exons (Figure 1A). Subsequently, an equimolar pooled fragment library was sequenced by Oxford Nanopore Sequencing. We then added all recorded variants in Ensembl (variant source dbSNP/ClinVar) (Howe et al., 2021) within the 5 fragments to the *LDLR* reference sequence [NG_009060.1 (O’Leary et al., 2016)] and mapped 50,000 randomly selected sequence reads to this reference sequence using the Minimap2 plugin (Li, 2018). Using the Geneious Variant caller, variants within the 5 fragments were identified and compared to the recorded variants, thereby differentiating between described variants with their dbSNP reference number and yet undescribed variants. The bioinformatic analysis was very fast taking in average 104±9 s for mapping and 19±3 s for variant calling.

To validate this workflow, a total of 4 patients were selected [2 related (father and son) and 2 unrelated] which had diagnosed FH with known disease-causing *LDLR* variants (Table 1; Figure 1B). For all samples, the coverage of the individual fragments was equally distributed, and the mean coverage was always above 900 (Figure 1C). In all cases, the variants were identified (Table 1). By analyzing the variant frequency, we could also determine the zygosity of the variants (heterozygous: 39%/48%, homozygous: 89%/92%). Analysis of the variant frequency from the two related patients (ID 2 + 3) permitted the clear distinction between heterozygous and homozygous inheritance pattern. We additionally discovered synonymous variants (2–5) in coding regions as well as a high proportion of intron variants (85–91%) without a reported clinical significance or with benign clinical significance. The majority of the variants found were recorded in Ensembl (72–86%). Because the analysis of 100,000 randomly selected reads did not significantly improve the variant calling results, we conclude that 50,000 reads per patient sample is sufficient for variant analysis.

All pathogenic variants had a strand bias between 52 and 91% and a *p*-value of 0 or close to 0. Because high strand bias values indicate a low confidence hit, we only included variants with a strand bias lower than 100% into the analysis excluding variants with a strand bias of 100% (Guo et al., 2012; Currin et al., 2019).

We noted a high amount of tandem repeat deletions/insertions among the found variants suspecting that this may be caused by sequencing errors. To test this hypothesis, we tested if tandem repeat deletions/insertions are found with a higher frequency in both a healthy control and a FH sample compared to all non-tandem-repeat variants. Fisher’s test yielded a p−value = 0.005044 indicating that tandem repeat deletions/insertions are caused by errors due to Nanopore sequencing. Consequently, we excluded all tandem repeat deletions/insertions from the further analysis.

### Identification of Unknown Pathogenic *LDLR* Variants

To test our workflow on patient samples with unknown pathogenic variants, we selected 3 patients from a single family [father (ID 7), mother (ID 5) and son (ID 6)] aged 6–40 years and 3 additional unrelated female patients (ID 8–10) aged 5–43 years with plasma LDL concentrations ranging from 5.8 to 18.7 mmol/L and suspected FH based on characteristic symptoms including xanthomas and a familial history of premature coronary artery disease.

**FIGURE 2:**
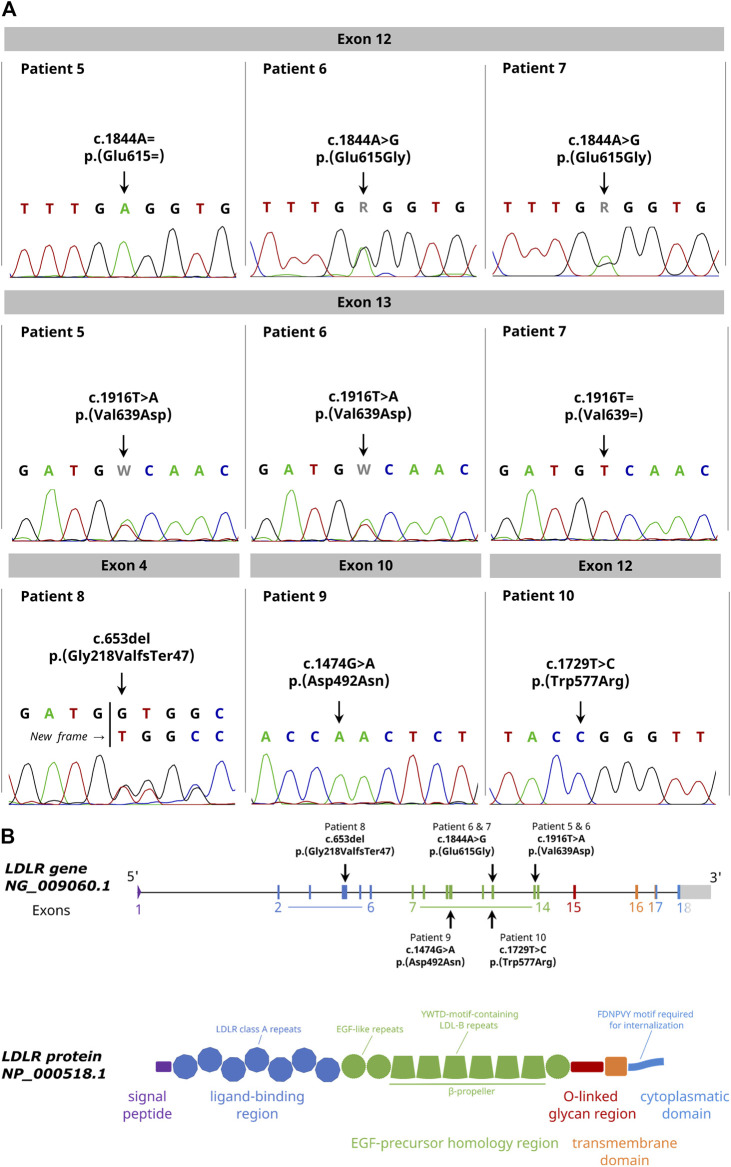
Detection of unknown pathogenic LDLR variants in FH patients with the Nanopore Sequencing workflow. **(A)** Sanger sequencing results confirming all identified variants with indicated change on DNA and protein level. **(B)** Graphical illustration showing the genomic and domain structure of the LDLR and the location of the identified variants within the *LDLR* gene. The exons and the corresponding protein domain are indicated by the same color.

We detected two heterozygous variants in the family. The father was carrier of the variant c.1844A > G p.(Glu615Gly) in exon 12 and the mother was carrier of the variant c.1916T > A p. (Val639Asp) in exon 13. Both variants were confirmed by Sanger sequencing. The zygosity agreed with the plasma LDL-C concentrations (patient 5: 5.81 mmol/L, patient 7: 6.46 mmol/L) indicating a heterozygous FH in the parents. Consistently with the high plasma LDL-C concentration of 13.52 mmol/L of the son, we identified a compound heterozygous inheritance pattern of both variants in the son (Table 2; Figure 2A). Notably, the variant c.1844A > G p.(Glu615Gly) of the father was not recorded in any public genetic database (Ensembl, gnomAD and Varsome) so far indicating the identification of a novel variant.

In patient 8, we found the heterozygous frameshift variant c.653del p.(Gly218ValfsTer47) in exon 4 confirmed by Sanger sequencing. This frameshift leads to a stop codon 47 codons downstream of the deletion resulting in a truncated protein which explains the higher plasma LDL-C concentration (9.59 mmol/L) compared to the two other heterozygous variant carriers (patient 5,7).

In the patients 9 and 10, we identified the homozygous variant c.1729T > C p.(Trp577Arg) in exon 12 and c.1474G > A p.(Asp492Asn) in exon 10. The zygosity of both variants in the patients was consistent with the high levels of plasma LDL-C (patient 9: 13.26 mmol/L, patient 10: 18.69 mmol/L)) indicating a homozygous FH.

Due to the prevalence of FH, high sample numbers are not expected in routine diagnosis. Therefore, the use of a single-use Flongle flow cell over a reusable flow cell is more rational. We could also identify the variants of patients 6, 7 and 8, when the samples were sequenced on a Flongle flow cell. Thus, our workflow is also attractive for the analysis of low sample numbers.

## Discussion

In this study, we describe a fast and easy feasible workflow for the genetic analysis of the *LDLR* gene using long-amplicon sequencing with rapid barcoding. Mutations in this gene contribute to 90% of all disease-causing variants in familial hypercholesterolemia (Humphries et al., 2006). Our workflow consists of the amplification of the promoter region and the coding sequence of all 18 exons *via* PCR, followed by sequencing using Oxford Nanopore sequencing technology and rapid bioinformatic analysis utilizing the software Geneious Prime. Nanopore sequencing offers several advantages over conventional sequencing technologies such as low cost, fast and easy sample preparation, no need of specialized laboratory requirements, parallel sample sequencing and analysis of long-read sequence libraries. Thus, this technique is an attractive approach for every diagnostic laboratory (Jain et al., 2016; Petersen et al., 2019).

Amplification of long amplicons enabled us to reduce the sequencing effort to the relevant regions by keeping the sample preparation effort to a minimum. Additionally, by mapping the sequence against the *LDLR* reference sequence instead of the chromosome 19 reference sequence or the whole human genome, we minimized the computing time. The subsequent use of the bioinformatic software Geneious Prime allows the bioinformatic analysis of the sequencing data without any programming skills. By adding all variants recorded in Ensembl within the 5 amplicons, we were able to rapidly match found variants to the recorded variants, thereby allowing rapid evaluation of the variants using the recorded information about clinical significance, variant consequence as well as SIFT and PolyPhen2 predictions (Adzhubei et al., 2010; Sim et al., 2012).

Mapping of raw read sequences against the *LDLR* reference sequence was performed using Minimap2 (Li, 2018). We found that 50,000 reads as input data yielded sufficient coverage (>900) identifying all known pathogenic variants. Because increasing the input data to 100,000 reads did not improve the variant calling, we conclude that using 50,000 reads per patient sample is sufficient for accurate genetic analysis. Analysis of the variant frequency allowed the distinct determination of the variant zygosity. We found variant frequencies of 39–48% for heterozygosity and 89–92% for homozygosity agreeing with another study considering variants with variant frequencies of 30–70% as heterozygous (Ahsan et al., 2021).

Strand bias is an indicator for variant calling quality. For the pathogenic variants analyzed, strand bias ranged from 52 to 91%. High strand bias values indicate low confidence calls meaning that the variant was found on either the forward or reverse strand with a much higher frequency. However, strand bias depends on the peripheral sequence around the variant and can differ between samples, thus filtering of the variant calls by strand bias should be performed with caution. To prevent the unintended exclusion of calls wrongly regarded as false-positive, we only excluded calls with a strand bias of 100% (Guo et al., 2012; Currin et al., 2019). There are two main modes leading to sequencing errors in Oxford Nanopore sequencing: certain sequences with indistinguishable conductance signals and irregular motor enzyme stepping (Noakes et al., 2019). This two modes may be sequence-specific (Currin et al., 2019) which may lead to differing sequences obtained from basecalling, and therefore account for varying strand bias of the variant calls.

Among the found variants, there was a high amount of tandem repeat deletions/insertions. We suspected this to may be caused by sequencing errors. Consequently, we tested if tandem repeat deletions/insertions are found with a higher frequency in both a healthy control and a FH sample compared to all non-tandem-repeat variants (Fisher’s test: p−value = 0.005044 ). This suggests that tandem repeat deletions/insertions are caused by errors due to Oxford Nanopore sequencing. Therefore, all tandem repeat deletions/insertions were excluded from the further analysis. It is reported by ONT that the determination of the correct number of bases of homopolymers is an issue with Nanopore sequencing (Brown, 2016). Consistent with other studies, we assume that tandem repeats lead to indistinguishable conductance states due to their inherent nature of repeating identical bases (Rang et al., 2018; Noakes et al., 2019). Additionally, these errors may be caused by sequence-dependent kinetics of the motor enzyme (Craig et al., 2017) complicating the resolution of tandem repeat length (Noakes et al., 2019; Whitford et al., 2021). Consequently, nanopore sequencing is reported to cause an increased frequency of homopolymer deletions (Cretu Stancu et al., 2017; Whitford et al., 2021).

Our study identified a novel variant in the *LDLR* gene which was not listed in the genetic databases (dbSNP, ClinVar, Ensembl, gnomAD and Varsome). A paternal missense variant c.1844A > G p.(Glu615Gly) in exon 12 was detected in a FH family with a compound heterozygous affected child. This variant is located in the EGF-like domain of the LDLR contributing to the receptor dissociation due to the lowered pH in endosomes (Figure 2B). Although, no clinical significance is reported for the identified variant, in the same codon, a c.1844A > T p.(Glu615Val) FH-causing variant has been previously reported and classified as likely pathogenic in ClinVar (Marduel et al., 2010). The in-silico mutation prediction tools PolyPhen2, SIFT, PROVEAN and Mutation Taster classified our novel p.(Glu615Gly) LDLR variant as pathogenic. Taken together, we conclude that the identified variant c.1844A > G p.(Glu615Gly) is likely to be pathogenic. In the same family, a maternal heterozygous missense variant c.1916T > A p.(Val639Asp) in exon 13 was identified, also located in the EGF-like domain of the receptor. The variant is reported with conflicting interpretations regarding pathogenicity in ClinVar. However, Nauck et al. (2001) report this variant as pathogenic and also published it in a family with FH.

We identified a heterozygous frameshift variant c.653del p.(Gly218ValfsTer47) in exon 4 in a female FH patient with angiographically proven single-vessel coronary artery disease. The variant is located in repeat 3 of the ligand-binding domain of the LDL receptor and leads to production of a shortened non-functional protein product (Giesel et al., 1995). Finally, our nanopore sequencing workflow identified the underlying homozygous variants in two girls (7 and 10 years old) with clinically proven homozygous familial hypercholesterolemia. In Patient 9, a missense variant c.1729T > C p.(Trp577Arg) was identified in exon 12. This variant lays within the highly conserved YWTD repeats that form the six-bladed ß-propeller domain of the LDLR and is known to produce a class 2 transport-defective LDL receptor (Soufi et al., 2009). In patient 10, a missense variant c.1474G > A p.(Asp492Asn) was detected in exon 10. This variant is also located within the highly conserved YWTD repeats of the six-bladed ß-propeller domain. However, this variant seems to produce a class 5 recycling-defective receptor (Galicia-Garcia et al., 2020).

Although the clinical relevance of genetic analysis of FH is being controversially discussed (Carlson, 2010; Aatre and Day, 2011), our results show that the identification of the underlying variants can have direct impact on the treatment. For example, the identification of the two homozygous YWTD repeat variants in patients 9 and 10 results in a class 2 and class 5 defect (class 2: transport defect and class 5: recycling defect). In our experience, homozygous carriers of these variants neither respond to statin nor PCSK9 inhibitor treatment, requiring LDL-apheresis. For these patients, the direct application of lipid apheresis would spare the patient by preventing unnecessary stress by ineffective treatment as statins can have severe side effects (Bełtowski et al., 2009). In contrast, patients 5–7 with variants affecting receptor dissociation outside of the YWTD repeats may benefit from statin medication as statin-mediated LDLR upregulation may compensate for the reduced receptor recycling. In case of patient 8 with the heterozygous frameshift variant and a higher LDL-C concentration compared to the other heterozygous patients, combination therapy of statin and PCSK9 inhibitors may be sufficient to reduce the LDL-C concentrations to the recommended level (Alonso et al., 2018). The additional inhibition of PCSK9 may increase the presence of the unmutated LDLR by decreasing the turnover rate of the receptor (Roth and Davidson, 2018). Additionally, the psychological effect of a confirming positive result may not be neglected. Studies reported that a definitive positive gene result helped the patients to accept the diagnosis potentially increasing patient’s compliance. Also, identification of the genetic cause often led to reassurance because by knowing that the relatives are either unaffected or can be early treated (Aatre and Day, 2011).

Here, we introduced a fast and easy PCR-based sequencing workflow for the rapid detection of pathogenic *LDLR* variants in the diagnosis of familial hypercholesterolemia. Through the use of Oxford Nanopore sequencing as a cost-effective method without the need for major system requirements and specialized laboratory equipment, our technique is an attractive easy-to-use tool for all diagnostic laboratories (Jain et al., 2016; Petersen et al., 2019). Identification of *LDLR* variants by traditional Sanger sequencing can be still challenging, costly and slow, especially when multiple patient samples need to be analyzed (Whitford et al., 2021). Also, other NextGen sequencing technologies for genetic diagnostics still are expensive (Petersen et al., 2019). For example, genetic analysis of the *LDLR* of one patient by Sanger sequencing would cost $1,060 and $890 by NextGen sequencing (PreventionGenetics, 2021). In contrast, considering the costs of DNA purification, PCR reagents, the Flongle flow cell and the rapid barcoding kit, the costs per sample minus labor are $109. Furthermore, the costs per sample depend on the number of samples sequenced by our workflow leading to a significant reduction if more than one sample is analyzed. So, for 3 patients (e.g., a patient and two family members), the costs per sample analyzed by Sanger sequencing are $1,060 ($3,180 in total) and $557 ($1,670 in total) when analyzed by NextGen sequencing (PreventionGenetics, 2021) while costs per sample analyzed with our workflow are reduced to $49 ($147 in total). Therefore, standard analysis of the patient’s family instead of the patient alone would be rational allowing early preventive treatment of affected family members.

Additionally, analysis using our workflow is notably faster and cheaper than analysis by Sanger or NextGen sequencing taking 18 days for standard order or 14 days for STAT order (PreventionGenetics, 2021). In contrast, upon arrival of the blood sample analysis of our workflow is completed within 3 days.

Finally, our workflow is not restricted to sequencing the *LDLR* gene alone but can be easily adapted to the other genes related with FH and most importantly, to any desired gene contributing to any hereditary disease by using our template documents (Supplementary Templates). We are currently adapting our workflow to the screening of genes associated with cardiomyopathy. Thereby, our workflow can be used to offer genetic diagnostics for a variety of clinically relevant hereditary diseases. Due to the technique of Nanopore Sequencing, these different samples can even be sequenced in the same run, thereby reducing the time and cost for the analysis. We believe that the workflow presented in this study is an attractive opportunity for every diagnostic laboratory to conduct fast and easy genetic diagnostics especially valuable for clinics to provide fast in-house genetic diagnostics.

## Data Availability

The raw data supporting the conclusion of this article will be made available by the authors, without undue reservation.
