# Supplementary material for: Fast and Easy Nanopore Sequencing Workflow for Rapid Genetic Testing of Familial Hypercholesterolemia
Source: Front Genet. 2022 Feb 9;13:836231. doi: 10.3389/fgene.2022.836231 (PMC8864071; doi:10.3389/fgene.2022.836231)
Supplement: Supplementary file 1 [file Table1.docx]

Supplementary Material

# Supplementary Data

Table 1: Oligonucleotides for amplification of the LDLR gene.

| **Fragment** | **Length**  **[bp]** | **Position** | **Primer ID** | **Sequence** | **T_m_**  **[°C]** |
| --- | --- | --- | --- | --- | --- |
| **1** | 1909 | 3592-5500 | LDLRPF | TCTGTCCAAGGCCGAATTCT | 64 |
|  |  |  | LDLR1R | AAAGAAGATGCGGTCCCTCA | 64 |
| **2** | 7414 | 15781-23194 | LDLR2F | GAAACGTGGTCAGTTTCTGATTC | 61 |
|  |  |  | LDLR6R | TGTCTCAGTCCCTTTCCTGG | 64 |
| **3** | 6480 | 26217-32696 | LDLR7F | AAGGGATGGGTAGGGGC | 65 |
|  |  |  | LDLR12R | CGTTCATCTTGGCTTGAGTG | 61 |
| **4** | 3394 | 35646-39039 | LDLR13F | AGAGGGTGGCCTGTGTCTC | 67 |
|  |  |  | LDLR15R | TGACCAAAATGTTCGTGGC | 60 |
| **5** | 3442 | 43572-47013 | LDLR16F | CATTTCTTGGTGGCCTTCC | 61 |
|  |  |  | LDLR18R | TCTCTGTCTTTGAATAAAACAAGGC | 60 |
