# Supplementary material for: Fast and Easy Nanopore Sequencing Workflow for Rapid Genetic Testing of Familial Hypercholesterolemia
Source: Front Genet. 2022 Feb 9;13:836231. doi: 10.3389/fgene.2022.836231 (PMC8864071; doi:10.3389/fgene.2022.836231)
Supplement: Supplementary file 2 [file DataSheet1.ZIP › Guideline.docx]

A Nanopore Sequencing Workflow

Complete Protocol

Amplicon Sequencing and SNP Calling

# PCR Setup

1. Design a PCR amplifying all exons of the GOI in several fragments (multiple exons can be amplified in one fragment, but large introns should be excluded). Short genes may be amplified in a single amplicon.
2. Optimize the PCR conditions to obtain a pure product with a low amount of unspecific products.

# Preparation of the Reference Sequence (example LDLR-Gene)

1. Download the reference sequence of your GOI at NCBI nucleotide (e.g., for the LDLR: NG_009060.1). Ensure that the file format is set to *Genbank (full)* and choose *Complete Record* and destination *File* in the export options.

If your GOI is reversed, ensure to select *Show reverse complement* under *Customize view/Display options.*

1. Identify the position of the first nucleotide of your reference sequence on the chromosome containing your GOI. This can be done by opening the whole chromosome sequence in BioEdit[[1]](#footnote-2) (e.g., for the LDLR: Chromosome 19 [NC_000019.10]) and by searching the first 10-20 nucleotides of the reference sequence in the chromosome sequence. Subtract 1 from the position and note down this number as it will be needed for conversion of chromosome into reference sequence positions and *vice versa*.
2. In your reference sequence, locate and note down the positions of the amplified fragments (e.g. in BioEdit[[2]](#footnote-3)). This can be done by searching the primer sequences in the reference sequence.
3. Convert the positions of the fragments in your reference sequence into chromosome coordinates:
4. Download all variants from Ensembl, which are located in your fragments using the chromosome coordinates of your fragments calculated in the previous step. For downloading this information from Ensembl, use the tool *BioMart[[3]](#footnote-4)* with the following configurations:

| **In the menu *Dataset* choose:** |
| --- |
| *Ensembl Variation 104* and  *Human Short Variants (SNPs and indels excluding flagged variants) (GRCh38.p13)* |
| **In the menu *Filter* check:** |
| Under *REGION:*  *Multiple regions* and enter the positions of your fragments in the format Chr*:Start:End:Strand*  e.g.: for fragment 1 of the LDLR: *19:11087972:11089880.*  Enter the position for each fragment in a new line. |
| Under GENERAL VARIANT FILTERS:  *Variant source: ClinVar* and *dbSNP* |
| **In the menu *Attributes* check:** |
| Under VARIANT ASSOCIATED INFORMATION:  By default, these should be already selected in *Variant information***:**  *Variant name*  *Variant source*  *Chromosome/scaffold name*  *Chromosome/scaffold position start (bp)*  *Chromosome/scaffold position end (bp)*  Check additionally:  *Variant alleles*  *Global minor allele frequency (all individuals)*  *Clinical significance* |
| Under GENE ASSOCIATED INFORMATION:  *Variant consequence*  *PolyPhen prediction*  *PolyPhen score*  *SIFT prediction*  *SIFT score* |
| Proceed by clicking on *Results* in the upper left corner and choose the option *Compressed web file (notify by email)* under *Export all results to* and set format to *TSV*.  Under *Email notification to*, enter your email address.  When your query is finished, you will be notified by email. Depending on the volume of your query, this may take a while. |

1. Download and install *R* and *RStudio*:

| *R* | <https://cran.r-project.org/bin/windows/base/> |
| --- | --- |
| *RStudio Deskop*  *(Free Version)* | [https://www.rstudio.com/products/rstudio/download/ - download](https://www.rstudio.com/products/rstudio/download/#download) |

1. Install the additional packages *dpylr* and *tidyr* by opening the R script file *install.R* and clicking the Source button.
2. Import the downloaded Ensembl BioMart file (.tsv) in R using the button *Import* and choosing *from text (base)*. Select the file that you have downloaded from BioMart.

In the next window, enter *Biomart_Data* as name and ensure that *na.strings* is set to *NA* and the option *Strings as factors* is unchecked.

1. Open the R script file “*vcf generation from Biomart Data.R”*. Under *#Enter chromosome start position*, replace the default number by from step 2 and source the script afterwards via the *Source* button. This should create a VCF file (usually in your personal Documents folder) containing all the downloaded variants.
2. If not yet installed, install the newest version (currently *2022.0.1*) of *Geneious Prime**[[4]](#footnote-5)*.
3. In *Geneious Prime,* create a new subfolder *Human* in the folder *Reference Features.* Import the reference sequence from step 1 into the newly created subfolder using *Add/Import files*.
4. When opening the imported reference sequence, several annotations should be visible in the *Annotations and Tracks* submenu. For display, only the annotation types *CDS*, *Exon*, *Gene* and *Misc feature* are necessary. Add a new annotation for each of your fragments by right-clicking anywhere in the sequence and selecting *Annotations/Add*. Enter the positions of each fragment on your reference sequence and choose the annotation type *Misc Feature*. Delete the default annotations in *Misc Feature* created during the reference sequence import.
5. Rename your reference sequence using the number of the chromosome of your GOI but save the original name for later. Select your reference sequence and import the VCF file created in step 8. Under *Tracks*, a track with all variants from Ensembl should appear. Rename the track *Ensemble Reference Variants.* Rename your reference sequence using the original name (i.e. the NCBI accession number).

# Data Analysis

1. Copy the raw data (.*fastq*) from your Oxford Nanopore device to your PC. Unzip the GZ files (e.g. with 7-Zip). Using the freeware *Merge Fasta[[5]](#footnote-6)*, merge all files per barcode into one file. Ensure that the output file type is *.fastq* by either setting the output file name to **.fastq* or manually renaming the file *.fastq.*
2. Next, randomly extract 50000 reads from the merged file using the freeware *FastaFastaQualConverter[[6]](#footnote-7)*. Alternatively, if you do not want to normalize the input, all reads can be used without the random extraction. We recommend renaming the file with a shorter name, e.g. *barcode1_50000_reads.fastq*.
3. Import the reads into *Geneious* using *Add/Import Files* and select *Oxford* *Nanopore* as *Read technology* (Creating a subfolder per sample prior to the import is recommenced). Ensure that *Don’t pair* is checked.
4. If not already installed, install the plugin *Minimap2* via *Tools/Preferences/Plugins and Features*. Right-click on the imported sequence list (
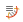
) and choose *Map to reference*. Under *Reference Sequence*, choose your reference sequence from chapter 2 step 11. Set all the rest like indicated in the figure below:


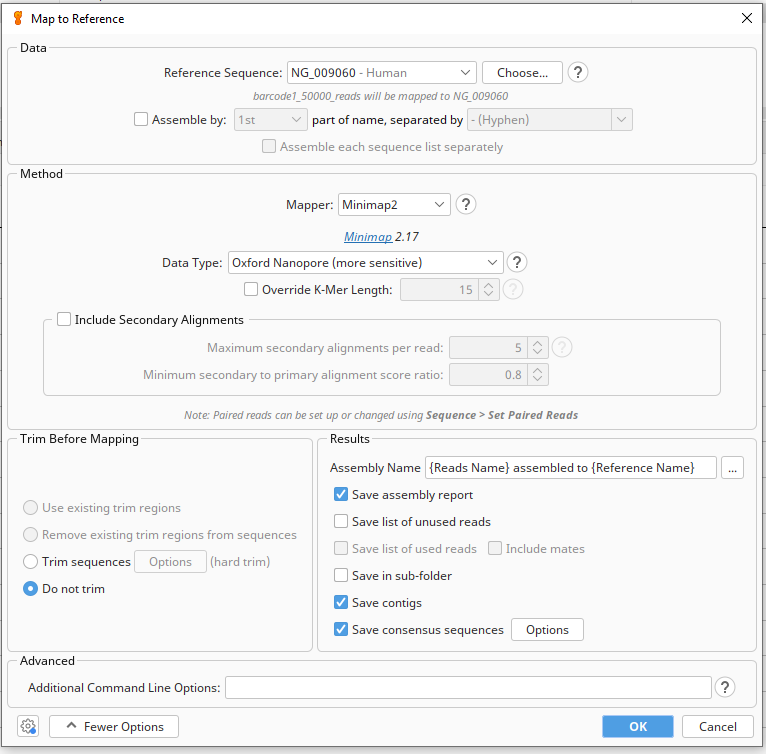


1. Select the newly created contig (
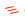
) and select all fragment annotations in the *Contig View*. Click on *Annotate & Predict* in the menu bar and select *Find Variations/SNPs.* The option *In selected regions only* should be checked automatically and should resemble the total number of your fragments. Set all the rest like indicated in the figure below:


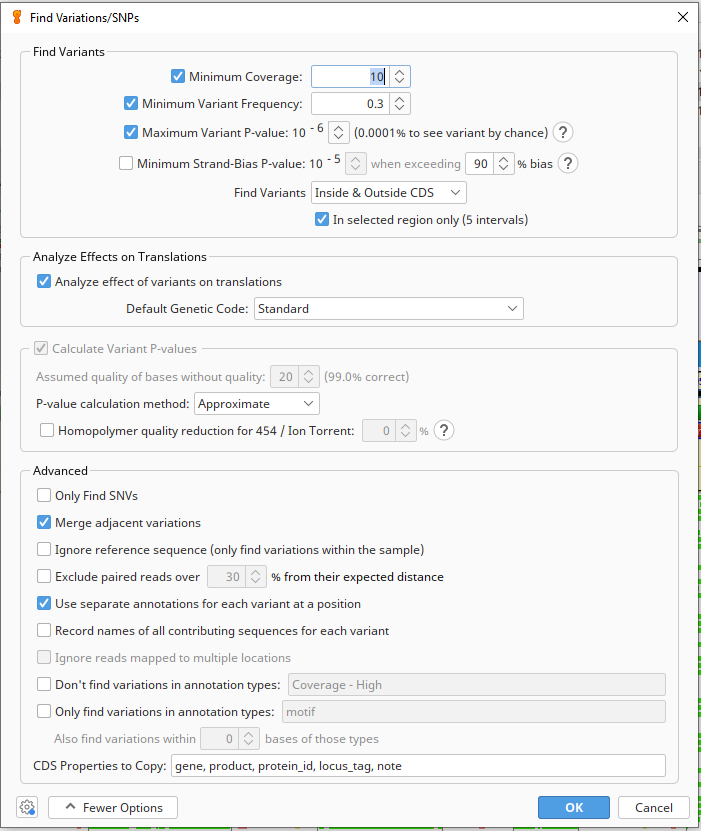


Under *Tracks,* a new track with all found variants will appear.

1. Click on *Annotate & Predict* in the menu bar and select *Compare Annotations.* In *Set A* select the newly created variant track and set all the rest like indicated in the figure below:


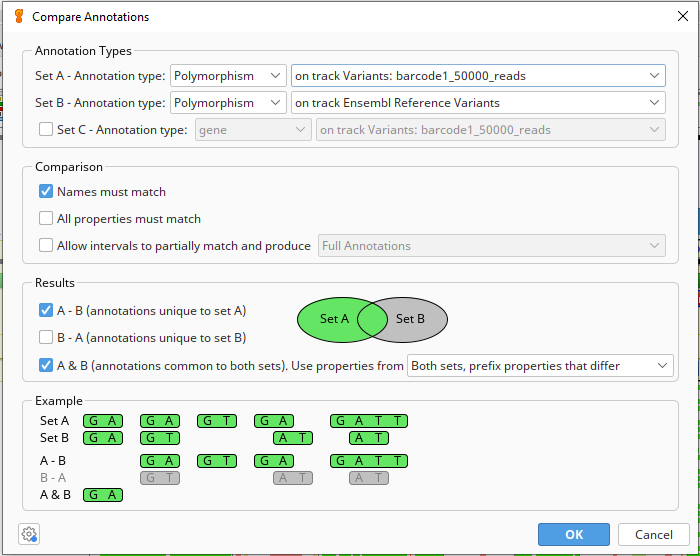


1. Two additional tracks will appear named “Name of Set A” **&** “Name of Set B” (referred to as &-track) and “Name of Set A” **-** “Name of Set B” (referred to as minus-track). The &-track resembles all variants, which are recorded in Ensemble; the minus-track resembles all variants, which are not recorded in Ensembl.
2. Open the contig by double-clicking and move to the *Annotations* tab. This will show a tabular view of the variants. Under track, choose the &-track. All found variants recorded in Ensembl will be displayed. To modify the information displayed, click on *Columns/Manage Columns* and add *CLIN_SIG, MAF, Variant Consequence* and *Variant ID*. Using Filter, filter the column *Strand-Bias* for variants, which are “<100”. Save the filter for further analysis by select *Saved Filters/Save current filter*.

If not already selected, also add the columns *CDS Position*, *Change*, *Reference Frequency*, *Variant Frequency*, *Variant P-Value*, *Strand-Bias*, *Amino Acid Change* and *Polymorphism Type*.

1. To show all variants, which are not recorded in Ensembl, choose the minus-track. If not already selected, also add the columns *CDS* *Position*, *Change*, *Reference* *Frequency*, *Variant* *Frequency*, *Variant* *P*-*Value*, *Strand*-*Bias*, *Amino* *Acid* *Change* and *Polymorphism* *Type*. Filter the column *Polymorphism Type* for variants, which not contain “*tandem*”. Save the filter for further analysis by select *Saved Filters/Save current filter*.
2. To look at a variant in the sequence in detail, select the variant in the annotation table and move back to *Contig View*. Zoom in with *Ctrl + mouse wheel*. Moving the cursor over the variant annotation will display a preview of the variant details. Clicking on the variant ID will open the variant in dbSNP.
3. A high-quality hit should have a *Variant P-value* very close to 0 or equal to 0, a *Strand-Bias* lower than 99 % and equal frequencies (≈ 38-50 %) for reference and variant allele (heterozygous) or a *variant frequency* greater than 80% (homozygous). For this analysis, focus on hits in CDS regions, which have a likely pathogenic or pathogenic *CLIN_SIG*.

1. BioEdit 7.2, <https://bioedit.software.informer.com/7.2/>, Accessed Dezember 6, 2021. [↑](#footnote-ref-2)
2. BioEdit 7.2, <https://bioedit.software.informer.com/7.2/>, Accessed Dezember 6, 2021. [↑](#footnote-ref-3)
3. BioMart, <https://www.ensembl.org/biomart/martview>, Accessed Dezember 6, 2021. [↑](#footnote-ref-4)
4. Geneious Prime 2022.0.1, <https://www.geneious.com/>, Accessed December 6, 2021. [↑](#footnote-ref-5)
5. Merge Fasta, <http://www.dnabaser.com/download/Merge%20Fasta/index.html>, Accessed December 6, 2021. [↑](#footnote-ref-6)
6. *FastaFastaQualConverter*, <https://www.mrdnalab.com/mrdnafreesoftware/fasta-qual-fastq-conversion.html>, Accessed December 6, 2021. [↑](#footnote-ref-7)
